# Development and Evaluation of a Psychosocial Intervention for Children and Teenagers Experiencing Diabetes (DEPICTED): a protocol for a cluster randomised controlled trial of the effectiveness of a communication skills training programme for healthcare professionals working with young people with type 1 diabetes

**DOI:** 10.1186/1472-6963-10-36

**Published:** 2010-02-09

**Authors:** Rachel McNamara, Mike Robling, Kerenza Hood, Kristina Bennert, Susan Channon, David Cohen, Elizabeth Crowne, Helen Hambly, Kamila Hawthorne, Mirella Longo, Lesley Lowes, Rebecca Playle, Stephen Rollnick, John W Gregory

**Affiliations:** 1 South East Wales Trials Unit, Department of Primary Care & Public Health, School of Medicine, Cardiff University, 7th floor Neuadd Meirionnydd, Heath Park, Cardiff, CF14 4YS, UK; 2 Department of Community Based Medicine, Bristol University, Belgrave Road, Bristol, BS8 2AA, UK; 3 Child Clinical Psychology Department, St Davids Hospital, Cowbridge Road East, Cardiff, CF11 9XB, UK; 4 Health Economics & Policy Research Unit, University of Glamorgan, Pontypridd, CF37 4BL, UK; 5 Bristol Royal Hospital for Children, Upper Maudlin Street, Bristol, BS2 8BJ, UK; 6 Speech & Language Therapy Research Unit, Frenchay Hospital, Bristol, BS16 1LE, UK; 7 Department of Primary Care & Public Health, School of Medicine, Cardiff University, 3rd floor Neuadd Meirionnydd, Heath Park, Cardiff, CF14 4YS, UK; 8 Nursing, Health and Social Care Research Centre, School of Nursing and Midwifery Studies, Cardiff University, Newport Road, Cardiff, CF24 0AB, UK; 9 Department of Child Health, School of Medicine, Cardiff University, Heath Park, Cardiff, CF14 4XN, UK

## Abstract

**Background:**

Diabetes is the third most common chronic condition in childhood and poor glycaemic control leads to serious short-term and life-limiting long-term complications. In addition to optimal medical management, it is widely recognised that psychosocial and educational factors play a key role in improving outcomes for young people with diabetes. Recent systematic reviews of psycho-educational interventions recognise the need for new methods to be developed in consultation with key stakeholders including patients, their families and the multidisciplinary diabetes healthcare team.

**Methods/design:**

Following a development phase involving key stakeholders, a psychosocial intervention for use by paediatric diabetes staff and not requiring input from trained psychologists has been developed, incorporating a communication skills training programme for health professionals and a shared agenda-setting tool. The effectiveness of the intervention will be evaluated in a cluster-randomised controlled trial (RCT). The primary outcome, to be measured in children aged 4-15 years diagnosed with type 1 diabetes for at least one year, is the effect on glycaemic control (HbA1c) during the year after training of the healthcare team is completed. Secondary outcomes include quality of life for patients and carers and cost-effectiveness. Patient and carer preferences for service delivery will also be assessed. Twenty-six paediatric diabetes teams are participating in the trial, recruiting a total of 700 patients for evaluation of outcome measures. Half the participating teams will be randomised to receive the intervention at the beginning of the trial and remaining centres offered the training package at the end of the one year trial period.

**Discussion:**

The primary aim of the trial is to determine whether a communication skills training intervention for specialist paediatric diabetes teams will improve clinical and psychological outcomes for young people with type 1 diabetes. Previous research indicates the effectiveness of specialist psychological interventions in achieving sustained improvements in glycaemic control. This trial will evaluate an intervention which does not require the involvement of trained psychologists, maximising the potential feasibility of delivery in a wider NHS context.

**Trial registration:**

Current Controlled Trials ISRCTN61568050.

## Background

Diabetes is the third most common chronic disease in childhood, with new cases affecting at least 13.5 per 100,000 children per year in the UK [1]. The incidence has doubled in the last 20 years. To reduce the risk of long-term complications associated with elevated blood glucose (e.g. retinopathy, nephropathy) effective treatment requires regular administration of insulin, most commonly by two to four injections daily in conjunction with a healthy lifestyle. The efficacy of management is monitored in the short-term by regular self-measurement of blood glucose concentrations and in the longer term by monitoring of glycosylated haemoglobin (HbA1c) levels in the blood and regular clinical review in paediatric diabetes clinics.

### Psychosocial aspects of diabetes

It is well recognised [2] that psychosocial and educational influences play a key role in determining management outcomes in children with diabetes. For example, a large audit in Scotland has shown that family structure is associated with glycaemic control throughout childhood [3]. During adolescence, rapid physical change (puberty) leads to relative resistance to the effects of insulin [4]. Concurrent major developmental changes include increasing independence, emerging sexuality and increased stress from peer and academic pressures. These factors together are often associated with deteriorating glycaemic control.

A recent NHS Health Technology Assessment (HTA) systematic review of the effects of educational and psychosocial interventions for adolescents with diabetes reported that there were no results from RCTs of such interventions in the UK [5]. However, the review did identify a study evaluating the effects of Motivational Interviewing (MI) on behaviour change in teenagers. The MI approach focuses on problem solving and goal setting to facilitate behaviour change, and emphasises the importance of using a guiding style of communication when consulting with patients. This trial was based on positive findings from a pilot study in children [6] and an RCT involving adults with type 2 diabetes [7,8]. The findings from this recently completed RCT of MI in children demonstrated persisting improvements in HbA1c up to two years after the start of a one year MI intervention when compared to a group receiving non-specific counselling [9]. Other psycho-educational interventions mostly in a North American context have produced small to medium-sized beneficial effects on a variety of diabetes management outcomes [10]. The HTA review concluded there is a need for well-designed clinical trials that recognise the inter-relatedness of various aspects of diabetes management and assess outcomes specifically targeted for change. In particular, the review recommended that such research be developed through a consultation process with stakeholders including patients, their families, healthcare professionals and health economists. Given the relative scarcity of trained clinical psychologists in paediatric diabetes services [11], this research project has been directed towards developing and evaluating a generic intervention that does not require delivery by trained psychologists. This requirement is emphasised by a more recent review [12] which identified a small number of UK-based studies of psychosocial interventions which have nevertheless been delivered largely by psychologists [6,9,13]. This review also described an ongoing UK evaluation of a family-based educational intervention in children and teenagers with diabetes [14,15].

### Behaviour change: what theory and research tell us

Theories of health behaviour change (e.g. reasoned action theory; the health action process approach) and the research associated with them have clarified the need to look beyond a simple approach to compliance and change based upon the delivery of expert information [16]. As Marteau and Lerman [17] have put it, "Just telling people they are at risk of developing a disease is rarely sufficient to change behaviour". Two variables run through many of the models as predictors of health behaviour change: beliefs about the value of change and beliefs about one's capacity to succeed (self-efficacy). The efficacy of theory-based interventions like cognitive-behaviour therapy have largely been attributed to their capacity to enhance self-efficacy [18]. Using a skills-based approach to counselling has been found to be effective in a number of fields [18,19]. So too, brief interventions have been found to be effective in facilitating change in a number of risky health behaviours [20].

A second line of research has focused on how the therapeutic relationship hinders or promotes motivation to change: an early effort to understand the effective ingredients of MI [21] identified a correlation between confrontational interviewing and resistance, and between "change talk" and behaviour change [22]. A recent meta-analysis of MI [23] found consistent evidence for effectiveness in some (e.g. alcohol, drug use) but not all behavioural domains. Interest in the application of MI in the field of diabetes among young people (predominantly adolescents) has also emerged [6,9,12,13,24]. One of the challenges in much of this research however, has been to specify exactly what elements of a complex method were used by the interventionists. It appears that some of the principles of MI can be realised in brief healthcare consultations, and that helping patients to clarify for themselves why and how they might change their behaviour can be more effective than brief advice-giving [25,26]. This body of work calls attention to both the direction of consultations about change (towards enhancing coping skills) and the way patients are spoken to (eliciting motivation and solutions from them).

### Intervention development

Development of the intervention, subsequently named the 'Talking Diabetes' programme, was informed by the Medical Research Council (MRC) framework for developing and evaluating (non-pharmacological) interventions [27,28]. A number of principles and conceptual aids guided intervention development. Firstly, there was the need to integrate talk about lifestyle change, self-control and quality of life with routine healthcare, where patients are at the receiving end of a range of medical and nursing interventions. Health-care staff need to find ways of moving between providing medical care on the one hand, and "letting go responsibility" on the other [8], to encourage children and teenagers to take control of their health, with assistance from others. During the intervention development stage, a consultative stakeholder reference group considered a consultation model which described the value of moving flexibly between directing, following and guiding *communication styles *when talking about behaviour change. A second conceptual and clinical challenge was the need to move beyond thinking about change as involving an isolated, single behaviour, a limitation in much of the theory of behaviour change in health psychology. The challenge was to help patients find a balance between multiple and inter-related health behaviours and lifestyle choices [5,7,8,29]. Thirdly, targeting or matching interventions to the needs of patients was a design consideration: efforts to match interventions with patients in other fields [30,31] have proved difficult. One approach to targeting was to regard this as something that happens not across interventions but within the consultation, as the practitioner shifts style and topic according to the needs of the patient [32]. To this end, there was some evidence for the acceptability and feasibility of using a targeting approach based on a flexible menu of strategies in which the practitioner and patient select a topic according to need [31,33]. This intervention framework has been developed from efforts to train healthcare practitioners to use elements of MI, and a recent application in drug misuse in young people has produced promising results [34]. In the present context however, it was not the intervention approach (MI) or content that was considered useful, but the use of a framework for targeting within the consultation based on a menu of topics for discussion.

Empirical and consultative work during the intervention development phase helped formulate and operationalise the 'Talking Diabetes' intervention. These activities included: (1) a review of psychosocial and educational interventions for children and teenagers with diabetes; (2) a telephone survey of practitioners (doctors, nurses, dieticians) working in UK paediatric diabetes clinics conducted to explore existing psychosocial practice; (3) a postal survey of UK practitioners exploring experiences and preferences for training in health communication skills [35]; (4) focus groups with young people with diabetes and with their carers; (5) observational work of consultations within three paediatric diabetes clinics in Wales and England; (6) experimental role-play with practitioners and young people; (7) an ongoing stakeholder consultation process built around three one-day workshops attended by teenagers with diabetes, their parents and multi-disciplinary health-care professionals with experience of managing children with diabetes, and (8) local piloting of approaches with young patients. The finalised intervention consists of a blended learning programme for practitioners in the paediatric diabetes field. The programme provides training in a number of communication strategies and skills to help practitioners prepare patients for behaviour change conversations and for conducting such consultations. A key element of this strategic approach was the development of a patient agenda-setting device (3T: TimeToTalk). These development activities will be reported separately.

### The current trial

The development stage provided evidence that the intervention is feasible for teams managing care, and is acceptable to patients and carers. A randomised controlled trial is now needed to test its effectiveness. The primary objective of the trial is to determine whether a multifaceted communication skills training intervention (incorporating a shared agenda setting component) for non-psychologist members of a paediatric diabetes team will improve clinical outcomes (HbA1c) for young people with type 1 diabetes. Secondary objectives include assessing intervention impact upon psychosocial outcomes (including quality of life), and assessing cost-effectiveness. A process evaluation will assess skill retention, competency and confidence of clinical team members in delivering the intervention.

## Methods/Design

### Ethical and governance approval

Multi-centre approval has been granted by Berkshire Research Ethics Committee (07/MRE12/9). Site-specific approval has been granted by local RECs at all trial sites and all participating Acute Trust Research and Development Departments.

### Design

**Figure 1:**
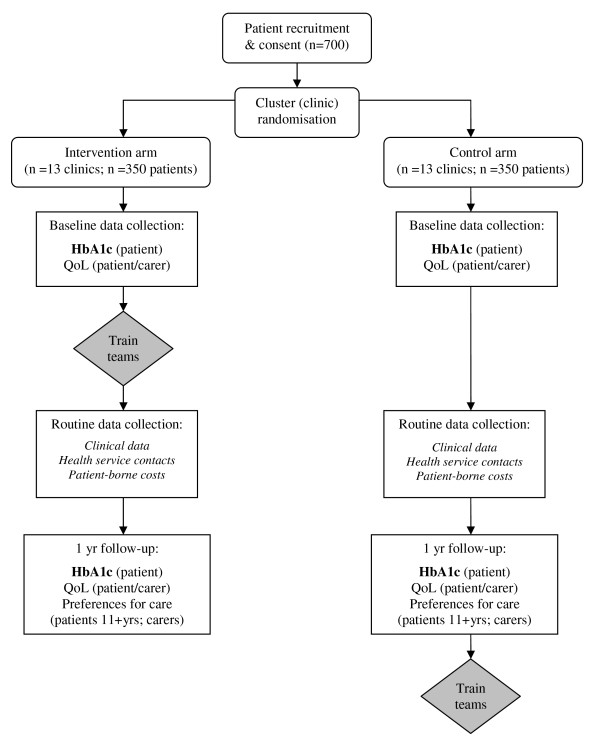
**Trial structure and outcomes**.

The study is a pragmatic cluster randomised controlled trial (Figure 1). Twenty-six teams will be randomised to receive training at the start (intervention group) or the end of the one-year study period (control group).

### Sample size

In order for an individually randomised trial to have 80% power to detect a moderate effect size of 0.4 for HbA1c at a 5% significance level, 200 patients would be required. Audit data from a Welsh paediatric diabetes interest group (The Brecon Group) relating to 750 children from all 13 centres in Wales indicates an intra-cluster correlation coefficient of 0.08 for HbA1c in patients aged 4 - 15 years. With 24 centres recruiting an average of 23 patients each, this inflates the total sample size required to 550. To allow for loss to follow-up, the intention is to recruit 700 patients (78% follow-up). Twenty-six centres have been recruited to allow for any subsequent centre drop-out.

### Clinic and patient selection

Potential clinics were approached using a variety of recruitment methods. Flyers outlining the nature of the study were distributed to members of the British Society for Paediatric Endocrinology and Diabetes (BSPED) and consultants and diabetes specialist nurses on a database compiled as a result of the surveys carried out during the development phase. Flyers were also distributed at professional and scientific conferences. Expressions of interest were received from 54 UK clinics. Thirty centres were approached to participate based on clinic size and geographical location: 26 agreed to take part and were able to meet contractual requirements.

All team members undergoing training will be consented prior to randomisation and the incentive of receiving training at the end of the study is provided to avoid differential levels of dropout or engagement between the two groups of teams. Recruitment bias is common in cluster randomised trials [36]. In this study, patients are identified and approached by a specially trained member of the local team (or occasionally a UKCRN research nurse) during the period prior to team randomisation and intervention training. All eligible patients are identified from clinic lists by the research nurse. A random sample of 40 patients will be selected by the coordinating study team (from an anonymised list) and approached en bloc by the research nurse, to obtain a target sample of 30 recruited patients per clinic.

### Inclusion and exclusion criteria

Each included clinic must be staffed by at least one paediatrician with an interest in diabetes, a diabetes specialist nurse and comprise of 40 or more potentially eligible children and adolescents. Eligibility criteria for participants are provided in Table 1.

**Table 1:** Participant inclusion/exclusion criteria

Inclusion criteria	Exclusion criteria
1. Type 1 diabetes	1. Not under care of parent or guardian (i.e. a looked after child)
2. 4-15 years old	2. Co-morbid chronic illness likely to impact on HbA1c independent of patient's ability to manage diabetes (e.g. condition requiring steroid treatment, cystic fibrosis, renal failure)
3. Under care of paediatric/adolescent diabetes team for duration of trial	3. In receipt of ongoing psychiatric/psychological therapy at the start of the study
4. Diabetes diagnosed > 12 months earlier	4. Other patients judged by clinical carer to be vulnerable due to existing medical or social condition
5. Parental or carer (and child when able) consent given	
6. Ability of patient and at least one parent or carer to complete study materials (questionnaires)	

### Recruitment

Participating families are recruited by the research nurse and written informed consent obtained from a parent. Where appropriate written informed consent or assent is obtained from the child (N.B. both parent and patient must be in agreement to take part in the study). Recruitment and randomisation of clinics was undertaken in three blocks [37]: however, patients within each centre will be approached en-bloc by letter.

### Randomisation

Half of the trial centres will be randomised to the intervention arm, and half to the control arm. Teams were recruited and then randomisation optimally balanced [38] for population (patient list) size. After the first bock of randomisations, each subsequent block incorporates the balance from the previous allocation(s).

### Trial procedures

#### Intervention

Members of clinical teams allocated to the intervention arm will undergo a blended training programme comprising web-based material and face-to-face seminars (The Talking Diabetes Programme). The training course aims to prepare practitioners for constructive behaviour change conversations with patients and to provide practitioners with strategies and skills for encouraging behaviour change. The training emphasises the importance of shared agenda-setting, and drawing upon the Motivational Interviewing approach, emphasises the importance of a guiding style when consulting with patients about behaviour change. Practitioners work their way through a number of distinct programme parts with an approximate total duration of 1.5 hours (delivered via three main e-learning modules). In addition, more interactive web-based components of the course allow practitioners to record their thoughts and experiences as they proceed through the programme. Two face-to-face seminars (approximately 2 weeks apart) with combined clinical teams also form part of the training course. Time spent on off-line learning activities such as discussing the training content in pairs, is recorded online. Following the second face-to-face workshop, participants will be invited to submit reports of three consultations in which they use their newly acquired skills and feedback will be provided by pre-assigned trainers.

The training programme shows practitioners how to use a device (3T: TimeToTalk) for promoting shared agenda-setting during clinical encounters with patients. This consists of a rigid folder and an inserted paper agenda pad of tear-off sheets which can be completed in advance by patients and carers to record topics of importance to be raised within consultations. Practitioners have the option to complete a proforma on which general topics discussed at clinic visits can be recorded and kept with patient notes, to facilitate clinical record keeping and communication between healthcare professionals. Copies of the paper agenda-setting pad (without folder) have been made available to each clinic to refill or replace folders as required and for patients not otherwise recruited to the study.

#### Frequency & duration of follow-up

Patients are required to provide blood samples, and patients and carers to complete questionnaires immediately post-recruitment, following their first clinic visit during the trial phase, and at one year. Professionals have their competencies measured post training and after one year to assess acquisition and maintenance of new skills. Professionals also provide attitudinal self-rating (importance and confidence) at the start and end of the training programme and at one year.

#### Baseline data collection

Baseline data is collected by the research nurse either in clinic or the patient's home. The research nurse provides patients and carers with a copy of an age-appropriate questionnaire (7-10 yrs; 11-15 yrs) assessing quality of life outcomes, to be returned directly to the trial coordinating centre (who will also follow up non-responders). The research nurse will complete a baseline Case Report Form (CRF), recording demographic information and clinical data (taken from patient notes).

Blood samples are collected by research nurses and sent to a single UK laboratory (Diabetes Research Network Wales Laboratory, Llandough Hospital) for measurement of HbA1c concentrations. Results are reported directly to the research team, following adjustment against the Diabetes Control and Complications (DCCT) international standard. Where a sample is lost or spoilt in transit, the research nurse will approach the patient and carer for consent to provide a second sample. In the event that a patient HbA1c sample is in excess of 15.0% (considered to be indicative of a patient at significant acute clinical risk) local diabetes teams responsible for patient care will be informed so that comparison can be made with the most recent HbA1c sample taken and analysed locally. Any patient contact resulting from notification of a high HbA1c value is at the discretion of the patient's diabetes care team: the research team have no direct contact with patients in connection with HbA1c. GPs will be informed in writing of their patient's participation in the trial by the research nurse.

#### Data collection during the trial phase: CRF and Interim Questionnaire

Clinical patient details (HbA1c, height, weight, BMI, insulin regimen), health service contacts and patient borne costs are recorded by the local research nurse at each clinic visit on the CRF. The research nurse also records who patients consulted with, for how long, and whether patients consulted on their own at each visit. Patients and carers are also asked to complete an interim questionnaire (assessing patient enablement) at their first clinic visit following the start of the trial.

#### Follow-up data collection

Capillary HbA1c samples for patients, and questionnaires for patients and carers will be repeated at one year. Where possible, primary outcome data (HbA1c) will be collected two weeks either side of the expected date of follow-up (i.e. within a 1-month window). Follow-up questionnaires will be sent to patients and carers directly by the trial coordinating centre. Follow-up questionnaires will also assess preferences for care using a Discrete Choice Experiment (DCE) not previously included at baseline.

### Primary & secondary outcomes

#### Patient outcomes

Careful selection of outcome measures for children is important to ensure appropriateness, feasibility and acceptability. Measure selection has been informed by two HTA systematic reviews [5,39] and through consultation with the stakeholder reference group in the intervention development phase. Patient-reported outcomes (assessed via an age-appropriate questionnaire at baseline and follow-up) include measures of diabetes-specific quality of life [40-42], self-efficacy [43], patient enablement [44] and patient perceptions of the diabetes team [43,45] importance of, and confidence in their ability to undertake diabetes care and monitoring activities (patients aged 11+ only) and preferences for care (DCE: follow up only [46]). Biochemical and clinical measures for patients include HbA1c, BMI, insulin type, dose and number of injections and self-reported frequencies of moderate and severe hypoglycaemic episodes.

#### Carer outcomes

Carer outcomes include demographic information (age, gender, ethnic origin, socio-economic status), parent measures of quality of life, anxiety and perceptions of the diabetes team [43] including items relating to communication between practitioners, feelings towards the next visit and continuity of care [45], importance of, and confidence in their ability to undertake diabetes care and monitoring activities. Proxy outcomes (patients aged 4-11) comprise diabetes-specific quality of life [40] and self-care [41]. Patient and carer outcome measures are summarised in Table 2.

**Table 2:** Patient and carer outcome measures

Outcome	Measure	Respondents
Diabetes-specific quality of life	Paediatric Quality of Life Inventory (PEDSQoL diabetes module: 11 items [40])	Patient (11+yrs), parent proxy (patients 5-11 yrs), parent
	Problem Areas in Diabetes (PAID: 23 items [41])	
	2 global items to assess change in QoL (follow-up only)*	

Perceptions of health care provider	Health Care Climate Questionnaire (HCC: adapted from 7 to 5-point scale [43])	Patient, parent
	3 additional items (respondents asked to circle an emotion to indicate anticipatory feelings towards going to clinic)*	
	Patient Enablement Inventory (PEI: 6 items [44])	
	Continuity of Care Scale (8 items [45])	

Self-efficacy	Perceived Competency Scale (4 items [43])	Patient, parent

Self-care	'Mis-management' (4 items adapted from Weisberg-Benchall et al. [42])	Patient (11+yrs), parent proxy (patients 5-11 yrs)
	Importance of self-care (6 items)* and confidence in ability (6 items)*	Patient, parent

*Indicates items created specifically for use in the current evaluation

#### Resource use

The cost of the intervention includes the cost of training intervention teams. The following training data will be recorded: travel costs to seminars, time spent on off-line learning activities (i.e. discussion of training content in pairs, reported on-line), time spent at seminars and time spent on-line (automatically recorded on website). Other training costs (e.g. venue, training materials) will also be calculated. Secondary costs are represented by between-group differences in service use including in-patient admissions (including ITU and HDU care), A&E attendances, clinic attendances, contacts with the diabetes team (home, telephone, face-to-face, electronic), other health service contacts (GP attendances, any other) and medication or equipment use (e.g. insulin type and dose). Patient-borne costs are also being assessed. These include travel to clinic, school absences and time taken off work by carers.

#### Process evaluation outcomes

Training outcomes for clinical teams will be evaluated and comprise behaviour change consultation competencies assessed via audio-recording of clinical sessions (post-training and at one year). Competencies for practitioners in the control group will be assessed at the end of the study period, prior to training [47]. Practitioners' confidence in, and importance of incorporating behaviour change into consultations will also be assessed (before and after training; one year after training); as will any systemic service changes at follow-up (e.g. timing of consultations).

### Analysis

#### Main analysis

The main analysis will be an intention to treat comparison of HbA1c values between the two groups of patients at one year. This will use multi-level modelling to allow for cluster (centre) and individual effects (including baseline concentrations of HbA1c as a covariate). Secondary analysis of other outcomes such as quality of life and cost will also be conducted using multi-level modelling incorporating baseline scores as covariates. A dose-response analysis will be conducted to explore associations between the amount of patient contact and an intervention effect. The two groups will also be compared on the non-attendance rate as the intervention may improve motivation to attend. A review of patient outcome measures used in diabetes, whilst predominantly in adults, concluded that whilst most have been shown to have content validity, there is less available evidence regarding reliability and responsiveness to change [48]. Responsiveness of the specific measures used will be assessed using both effect sizes and correlation to clinical variables and self-rated change.

Competence, confidence and importance of behaviour change counselling will be compared between the two groups at one year using a two level linear regression model controlling for profession. Short and long term impacts of the intervention will be analysed within the intervention group only using repeated measures ANOVA. Analysis of reliability of the competence inventory will be conducted on the trial data.

No formal subgroup analyses are planned. However, exploratory analysis of the impact of patient level factors (e.g. age and gender) and clinic level factors (e.g. size of clinic) on the effect of the intervention will be carried out. No interim analyses are planned.

#### Economic evaluation

A cost effectiveness analysis is assessing total costs against the primary outcome (HbA1c) and will be reported in the form of an incremental cost effectiveness ratio [49]. The economic element of the study also involves the assessment of preferences for delivery of care, and will be assessed using a Discrete Choice Experiment (DCE) to be administered as a separate questionnaire at one year only. A DCE works by presenting individuals with hypothetical scenarios involving different *levels *of defined *attributes *and asking them to make discrete pair-wise choices [46]. During the development phase of the current study the patient and parent focus groups and the stakeholder reference groups were used to identify the most relevant attributes of diabetes care and to clarify levels. This developmental work will be described separately. In the trial phase, the DCE will be administered to patients aged 11 and above, and carers of all patients, at follow up. Possible differences between children's preferences and those of their carers will be explored.

## Discussion

Psychosocial factors are well-known to have a significant impact on the effective management of young people with diabetes in addition to medical management. Research which takes into account the inter-relatedness of these aspects of successful management is therefore required, in particular evaluations of interventions developed in consultation with key stakeholders which are designed to target these psychosocial influences.

The primary objective of the current trial is to determine whether a training package developed in consultation with healthcare professionals, patients and carers, for staff working in paediatric diabetes services to help them engage their patients with behaviour change, results in significant improvements in clinical outcome (HbA1c) for young people with type 1 diabetes. Previous research indicates the effectiveness of psychological interventions in achieving sustained improvements in glycaemic control. However, the current trial, based on the principles of MI and behaviour change theory, is the first to evaluate the effect on HbA1c of an intervention in children, based on these principles but not requiring the involvement of a trained psychologist. Clinical psychology services are currently inadequate in paediatric diabetes care: such an approach therefore allows these limited resources to be directed where most needed, maximising the potential feasibility of intervention delivery across the NHS. The effectiveness of the intervention in terms of quality of life for patients and carers, and cost-effectiveness will also be evaluated.

The current trial does however pose a number of challenges, perhaps most notably relating to skill maintenance on behalf of the diabetes care teams. Competency assessment will therefore be carried out immediately after training, and at the end of the one-year trial period for intervention teams, and immediately prior to training for control teams. Challenges common to cluster randomised trials generally include the potential for recruitment bias. However, in the current study all patients are identified and approached by the research nurse during the period prior to team randomisation and intervention training. Clinical teams are also consented prior to randomisation and the incentive of receiving training at the end of the study is provided to avoid differential levels of drop-out or engagement between the two groups of teams. Currently 26 centres have been recruited to allow for some subsequent drop-out. To allow for participant loss to follow-up, 700 patients (approximately78% follow-up) will be recruited. The broad entry criteria also mean that the majority of each centre's patient group will be eligible and enhances the generalisability of the trial results.

It is expected that the current trial will add to the growing body of evidence for the effectiveness of psychosocial interventions in improving psychological and clinical outcomes for children and teenagers with type 1 diabetes. Additional aims of the study are to demonstrate both feasibility in terms of roll-out across the NHS and cost-effectiveness, given that the intervention is the first of its kind not requiring specialist input from clinical psychology services, which are currently limited in paediatric diabetes care.

## Competing interests

The authors declare that they have no competing interests.

## Authors' contributions

JG and MR were co-principal investigators and guarantors of the study in its entirety. JG, MR, KeH, KB, SC, DC, LC, HH, KaH, ML, LL, RP, SR and RM were responsible for developing the research question and study design, and implementation of the study protocol. RM was responsible for trial management. RM, JG, MR and KH were responsible for drafting the manuscript. All those listed as authors were responsible for reading, commenting upon, and approving the final manuscript.

## Pre-publication history

The pre-publication history for this paper can be accessed here:

http://www.biomedcentral.com/1472-6963/10/36/prepub
